# Cerebrovascular Disorders: Role of Aging

**DOI:** 10.1155/2012/128146

**Published:** 2012-03-19

**Authors:** Aurel Popa-Wagner, Ana-Maria Buga, Ryan C. Turner, Charles L. Rosen, Emil Toescu

**Affiliations:** ^1^Department of Neurology, Clinic of Neuology, Aging & Neural Repair Group, Medical University of Greifswald, Ellernholzstrasse 1-2, 17489 Greifswald, Germany; ^2^University of Medicine and Pharmacy, 200344 Craiova, Romania; ^3^Department of Neurosurgery, West Virginia University, One Medical Center Drive Morgantown, WV 26506, USA; ^4^School of Clinical and Experimental Medicine, College of Medical and Dental Sciences, University of Birmingham, Vincent Drive, Edgbaston, Birmingham B15 2TT, UK

Therapeutic development for neurological diseases, whether cerebrovascular disease, vascular dementia, or Alzheimer's disease (AD), has been largely unsuccessful. Despite the apparent success of numerous pharmacologic agents in preclinical animal models, few have translated successfully from bench to bedside. As such, a vast need remains unmet and additional investigation is required.

Speculation concerning the reasons for this failed translation from bench-to-bedside often highlights either the lack of reproducibility or a poor choice of the outcome measure parameters. However, in most of the literature, the lack of clinical relevance of the animal model used often goes unnoticed. The role of age in neurologic disease development is well known, in fact, age is the greatest risk factor for development of AD as well as stroke. In sharp contrast to this clinical reality, the vast majority of preclinical studies utilize young or young-adult animal models of neurologic disease, despite the suggestion of collaborative advisory groups such as the Stroke Therapy Academic and Industry Roundtable (STAIR).

The ageing process affects various biological axes, from the status of the cardiovascular system to the status of the inflammatory responsiveness and finally to the changes in the ability of the neural systems to handle pathological insults and a significant reduction in the recovery capability. At one end of this spectrum of changes in cerebral cardiovascular status are the morphological changes in vasculature, manifested as large increases in the vascular path due to increased tortuosity of arterioles in the deep white matter [1] and an age-associated decrease in capillary number and length, leading to a significant increase (25%) of the intercapillary distance in both the hippocampus and cortex [2] and white matter [3]. Such processes would lead to tissular hypoxia that in young or adult brain would trigger an angiogenesis response, mediated by HIF-1 as a transcription factor for VEGF. However, in the aged brain, there is a reduction in the angiogenesis response, due to decreased responsiveness to HIF-1. Such age-associated changes in the blood vessels architecture are even more relevant to the brain, because of the specifics of vascularization: the system of feeding arteries is situated near the surface of the brain, from where end-arteries are penetrating through the gray and white matter in a network that terminates in a capillary bed but with very few shunts [4].

As a result of such anatomical changes, there is little surprise that in the aged brain there is a significant reduction in the cerebral blood flow (CBF), affecting mainly the cortex, and more sparingly the subcortical regions, as revealed by a variety of imaging techniques, from positron-emission tomography (PET) and single-photon emission computed tomography (SPECT) to high-resolution, contrast-enhanced MRI and arterial-spin labeling (ASL) [5]. However, as it has been pointed out [6], CBF is affected not only by morphological changes, but it is also modulated by a variety of functional parameters such as perivascular innervation [7], astroglial control of arteriolar constriction [8], and autocrine endothelial signaling in response to rheostatic forces and neural environment [9].

For the clinical category of cerebrovascular diseases, the stroke is, by a distance, the most significant entity, both in terms of prevalence and consequences. The stroke can result from either an occlusion of the vessel (ischemia), which can be either transient (e.g., the transient ischemic attack, TIA) or of longer duration; or from the rupture of a vessel, leading to regional hemorrhage, either within the cortical matter or in the dural cavities. While the biological and medical consequences of a stroke are significant at any age, the incidence and the severity of a stroke are significantly increased with age. The paper from Russo et al. provides a systematic review, looking at the first stroke incident and shows that the trend continues for the very old (older than 80 years old).

Several factors are likely contributors to this increased severity of stroke with age. One such factor is a metabolic decrease in the capacity of neural cells to counteract extreme stressors and/or neurotoxic challenges (decreased metabolic reserve) [10] that appear in the penumbra region of a stroke, in which a combination of hypoxia and resulting decrease in ATP provisions will lead to the generation of a hyperexcitable environment, posing significant metabolic challenges.

Stroke in the very old is a very frequent condition, with an unfavourable outcome, and makes a relevant contribution to the social burden of stroke and may require more efficient, dedicated stroke services (Russo et al.). Aged individuals recover less well from stroke, and rehabilitation aims at improving the physical and cognitive impairments and disabilities of patients with stroke. Therefore, studies on behavioral recuperation after stroke in aged animals are necessary. Various experimental settings have been used to assess the recovery of sensorimotor functions, spontaneous activity, and memory after ischemia in aged rats. Overall, the results indicate that aged rats have the capacity to recover behaviorally after cortical infarcts, albeit to a lesser extent than their young counterparts [11, 12]. It should be kept in mind, however, that before stroke aged rats are already impaired as compared to young animals and show significantly decreased performance in some tests like spontaneous activity [13]. Accelerated glial reactivity to stroke in aged rats correlates with reduced functional recovery [13] including Morris water-maze [14]. Behavioral and histological effects of chronic antipsychotic and antidepressant drug treatment in aged rats with focal ischemic brain injury are discussed in [14].

Another crucial component of the brain tissue response to stroke is the inflammatory response. As reviewed in the article by Shah and Di Napoli (2011) the age-associated changes in the immune function determine that in the aged and individuals, the response to an acute stroke involves a more intense inflammatory reaction in the first phase of acute ischemia, involving cytokine activation and chemokine expression that lead to an early scar formation and fibrosis. In agreement with previous observations [15], the article also points out the fact that the biology of the aged brain is different from that of the young brain, and this has significant implications for the current translational efforts to define effective therapies, since a large majority of interventional studies are performed on young and young-adult animals.

Another important perspective in asserting new potential therapeutical interventions for stroke is to assess if the differences in responsiveness between young adult and old brains are due only to functional changes or if they involve more elaborate changes in protein expression. Recent detailed analyses of the genomics data from adult and aged animals indicate changes in the balance of the various systems and regulatory processes. Using categorized DNA arrays, we found inappropriate gene regulation in response to stroke both in the ipsilateral and the contralateral hemisphere of aged rats. The gene expression profile in the brains of poststroke aged rats is indicative of increased cell death due to DNA damage and apoptosis, especially in the first week after stroke. Similarly, we reported persistent downregulation of genes that are required for neurogenesis after stroke in aged rats [16].

However, the category of cerebrovascular diseases is not restricted to the pathologies involving the large vessels. Cerebral small vessels disease (cSVD) is a group of pathological processes associated with established vascular risk factor such as hypertension, atherosclerosis, diabetes mellitus, and atrial fibrillation. The consequences of small vessel disease on the brain parenchyma are mainly lesions located in the subcortical structures such as lacunar infarcts, white matter lesions, large hemorrhages, and microbleeds. This compendium of tissue lesions has been compiled in the last decade or so by the ever expanding use of more powerful and sensitive brain imaging technologies, rather than by a better understanding or direct assessment of actual small vessels territories. As a result, the cerebral small vessels diseases are now invoked in explaining a variety of clinical syndromes, and van Uden and collaborators discuss in their paper the possibility that such vascular pathology is underlining the clinical manifestations of the late onset depression in the aged people (I. W. van Uden et al. (2011)). Even more daring, Teggi et al. propose that cSVD might also explain some forms of the Meniere disease [17].

Another area of neuropathology in which small vessel disease has a well-established role that gathers ever increasing recognition in both the clinical and basic research world is that of being a leading cause of cognitive decline and functional loss in the elderly. Vascular dementia is a broad concept that has evolved slowly. While general anecdotal references linking putative cerebral vascular events with reductions in cognitive performance have existed for long time, it is the articles of Binswanger and Alzheimer around the turn of the 20th century describing in detail the various types of vascular lesions that could be encountered in human brains that set the field on a strong morphopathological footing, and it took another 70 years until Hachinski suggested that dementia of vascular origin was a consequence of multiple strokes [18]. Subsequently, the concept continued to evolve to include multiple mechanisms related to deficiencies in cerebral blood supply, including large vessel disease, small vessel pathology, consequences of cerebral hypoperfusion and hemorrhage.

While the risk factors for vascular diseases are relatively well established, much less consensus exists on the specific risk factors for vascular dementia, that is, apart, from the acknowledged role of increased age. Ongoing interest in cerebrovascular diseases research has provided data showing that Alzheimer's proteins and other factors may be involved in the pathogenesis of gradual ischemic brain injury. Thus, both focal and global brain ischemia in rodents produce a stereotyped pattern of selective neuronal degeneration, which is just the same as in Alzheimer's type dementia. As hypothesized in the article by Enciu et al., there is an overlap of events between chronic hypoxia and AD on several levels, such as hypoxic-triggered cellular pathways, inflammatory environment, growth factor signaling, and calcium homeostasis. A reduction of CBF and a series of molecular events precede the major ischemic events in vascular cognitive impairment. Based on these subtle changes, intervention at early stages could prevent the full-blown development of dementia, which might represent a “point of no return” for the neurovascular units and neuronal networks with few chances for efficient treatment. In their extensive review, Enciu and her colleagues also discuss some of the molecular mechanisms that are triggered by the cerebrovascular diseases and that lead to overcognitive dysfunction and argue convincingly the case for an early intervention at the point at which the dysfunction of the neurovascular units is still potentially reversible.

However, it is important to keep in mind that the genetic makeup is a hugely important factor, from the subtle alterations in the function of various proteins (e.g., ApoE4) to the overt cerebrovascular diseases with a genetic control, as the cerebral autosomal arteriopathy with subcortical infarcts and leukoencephalopathy (CADASIL) that involves mutations in Notch-3 protein. Equally important is the discovery that fundamental genetic changes may occur with aging, such as the CLOCK genes, as discussed by Thome et al. (2011). Taken together, aging may result in fundamental changes throughout the body, beginning at a gene level and progressing to protein expression or posttranslational modification level.

It is clear that the quest for improved therapeutics will require increased understanding of disease pathophysiology and, in particular, the changes induced by the aging process. Similarly, neurologic disease research has often maintained a “neurocentric” focus, in which the role of the neuron was emphasized. This is unlikely to result in successful development of therapeutics due to the intimate relationship evident between neurons, surrounding glia, and the neurovascular unit—in both health and disease. The effect of aging on astrocyte and microglial response to injury and how this process can be manipulated successfully for therapeutic development must be considered.

Similarly, preclinical studies of neurologic disease utilizing aged animals must incorporate functional outcome measures in addition to histological measures. Clinically, the primary assessment remains functional outcome and presence/absence of significant disability yet preclinical studies often emphasize histological outcomes such as infarct volume. The difficulty of identifying high fidelity functional studies in aged animals remains a vexing problem.
